# Analysis of winter diet in Guizhou golden monkey (*Rhinopithecus brelichi*) using DNA metabarcoding data

**DOI:** 10.1002/ece3.10893

**Published:** 2024-02-01

**Authors:** Xu Zhang, Huafu Zhong, Jingcheng Ran, Jiaxin Luo, Meifeng Chen, Haibo Li, Yeying Wang, Shaochuan Cheng, Yuying Yan, Xiaolong Huang

**Affiliations:** ^1^ Guizhou Academy of Forestry Science Guiyang China; ^2^ Guizhou Fanjingshan Observation and Research Station for Forest Ecosystem National Forestry and Grass‐land Administration Tongren China; ^3^ Guizhou Caohai Observation and Research Station for Wet Ecosystem National Forestry and Grassland Administration Bijie China; ^4^ Key Laboratory of National Forestry and Grassland Administration on Biodiversity Conservation in Karst Mountainous Areas of Southwestern China Guizhou Academy of Forestry Guiyang China; ^5^ Fanjingshan National Nature Reserve Administration Tongren China; ^6^ College of Life Science Guizhou Normal University Guiyang China

**Keywords:** colobine, diet composition, endangered species, grey snub‐nosed monkey, montane broad‐leaved forest habitat, presbytini

## Abstract

The Guizhou golden monkey (*Rhinopithecus brelichi*) is a critically endangered wildlife species, and understanding its diet composition may be useful for assessing its feeding strategies. DNA metabarcoding was used to determine the dietary diversity of *R. brelichi*. DNA was extracted from 31 faecal samples and amplified chloroplast *rbcL* and mitochondrial *COI* DNA was sequenced using the Illumina NovaSeq platform. A comparative analysis of the sequences revealed that the five most abundant plant genera were *Magnolia*, *Morinda*, *Viburnum*, *Tetradium* and *Eurya*. In winter, *R. brelichi* mostly consumed shrubs, herbs and shrubs/trees according to the habit of plant genera with higher abundances comparatively. The five most abundant families in animal diet were Psychodidae, Trichinellidae, Staphylinidae, Scarabaeidae and Trichoceridae. This study is the first to show the composition of the winter animal diets of *R. brelichi* based on DNA metabarcoding. These results provide an important basis for understanding the diet of wild *R. brelichi*, which inhabits only the Fanjingshan National Nature Reserve, China.

## Graphical abstract

Our DNA metabarcoding analysis has revealed that *Rhinopithecus brelichi* predominantly feeds on plants from the Magnoliaceae and Rubiaceae families, Diptera and Coleoptera insects, as well as macrofungi such as Agaricales throughout the winter season.

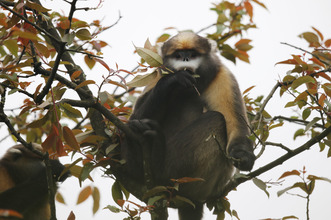

## 1 INTRODUCTION

**FIGURE 1:**
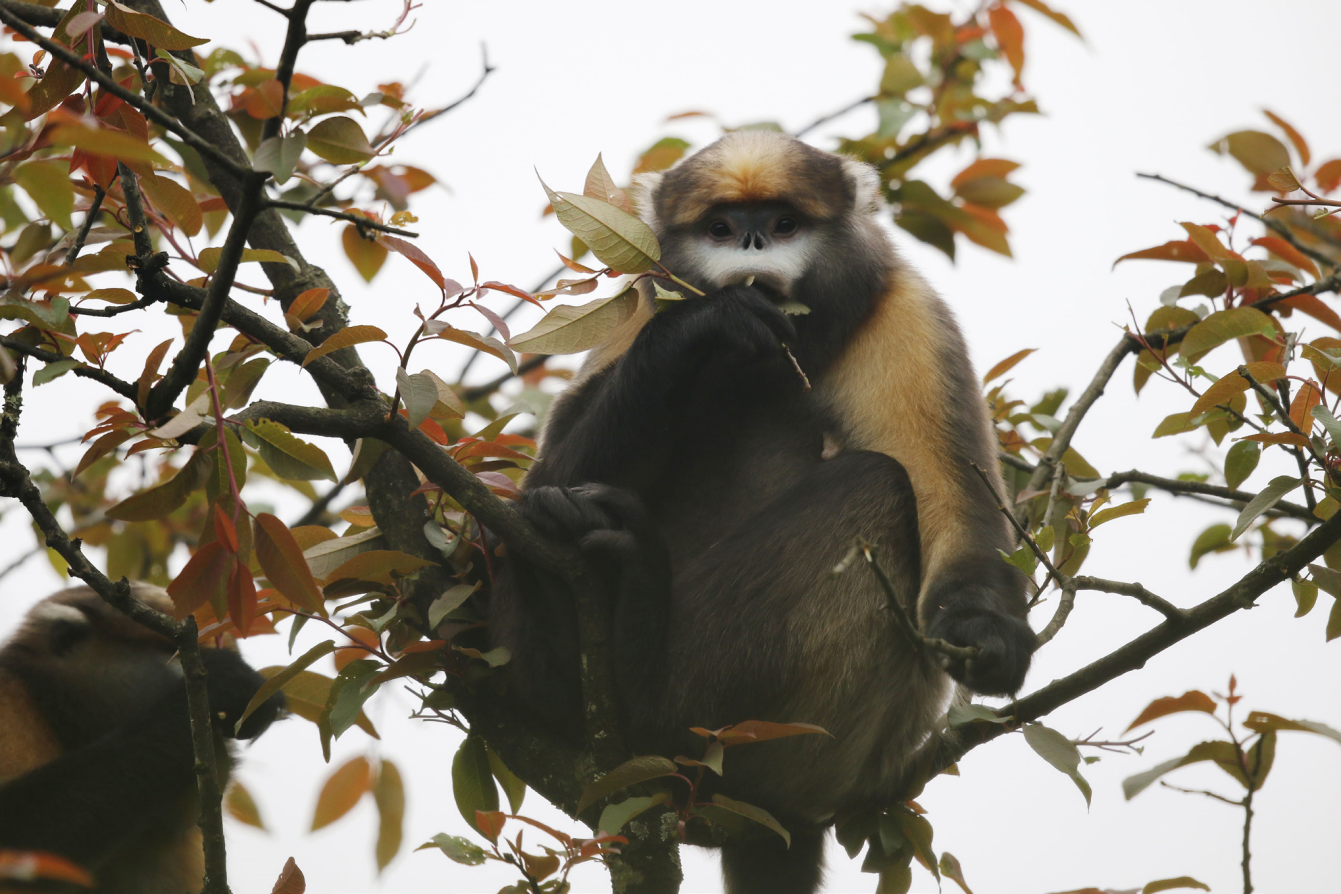
*Rhinopithecus brelichi*. A Guizhou golden monkey is eating the leaves of *Prunus obtusata* with a companion. The photograph was taken by J.C. Ran.

The Guizhou golden monkey (*Rhinopithecus brelichi*) (Figure 1), also called the grey snub‐nosed monkey, is one of the world's critically endangered primates (Long et al., 2022; Mittermeier et al., 2022) and is found only in the Fanjingshan National Nature Reserve (FNNR), which encompasses 419 km^2^ in China. *R. brelichi* belongs to the genus *Rhinopithecus*, which belongs to the group of odd‐nosed monkeys of the colobines in the family Cercopithecidae (order Primate). *R. brelichi* is phylogenetically closest to *R. roxellana*, which diverged approximately 1.01 million years ago (mya) (Yu et al., 2016). *R. brelichi* is smaller than *R. roxellana* and has a grey‐blue face. The grey snub‐nosed monkey is grey overall, with golden hairs growing on its shoulders, upper arms and top of its head. In the 1960s, *R. brelichi* was mainly found at 500 m above sea level, and by the 1980s, it was found mainly at 800 m and above (Quan & Xie, 2002), with recent reports indicating that the main range of *R. brelichi* is now 1200–2100 m above sea level (Long et al., 2022). Wu et al. (2004) suggested that temperature and food availability are the main factors determining the range of *R. brelichi* and that altitude, temperature and vegetation are the major factors limiting habitat selection for *R. brelichi*.

Diet analysis is a prerequisite for predicting animal survival, social organization and the relationship between animals and their environment (Liu et al., 2021; Zhao et al., 2020). The quantity, quality, spatial distribution and temporal variability of food resources are important factors influencing primate diet, behaviour, reproduction and groups, etc (Foerster et al., 2016; Osman et al., 2020; Strier, 2021). High‐quality foods rich in easily digestible energy and protein are often preferred for primate feeding (Strier, 2021). Furthermore, fibres, minerals and secondary compounds in plants, such as alkaloids, tannins and phenolics, also influence primate food choice and behaviour (Zhao et al., 2020). However, the availability of high‐quality food is temporally and spatially limited in the wild, making fallback foods particularly important when food is scarce for primates (Strier, 2021). Methods traditionally used to analyse wildlife food habits include observation of foraging behaviour, stomach contents, faecal microscopy and stable isotope analysis (Sterling et al., 2013). Rare, endangered and elusive animals are difficult to observe in the field, so faecal samples are important because they can provide ecological information even without direct observation (Srivathsan et al., 2015). Faecal microanalysis is demanding microscopic identification, labour intensive, and has low resolution for dietary items with similar micromorphological organization (Liu et al., 2021). DNA metabarcoding techniques, which have high sensitivity and high data throughput, can be used to analyse the composition of faecal samples to allow for finer taxonomic detail, especially for omnivores.

DNA metabarcoding, which combines high‐throughput sequencing (HTS) with DNA barcoding, allows for the processing of a greater number of samples and the recognition of multiple taxa in a shorter amount of time (Drake et al., 2023; Kuang et al., 2023). Because of its advantages, DNA metabarcoding has been used in dietary studies of a variety of animals, including sharks (Olin et al., 2023), *Lutra lutra* (Drake et al., 2023), birds (Schumm et al., 2023) and others. Accordingly, DNA metabarcoding of faecal samples has been used to characterize the plant diet of primates. The species composition of the plant diet of *Macaca arctoides* was identified (Osman et al., 2020), changes in the plant diet of *Chlorocebus pygerythrus* were detected during the dry and rainy seasons (Brun et al., 2022), and guidelines for habitat management were provided (Osman et al., 2022). Moreover, DNA metabarcoding has been used for identifying the animal component of primate diets. For instance, Hamad et al. (2014) reported that African great apes consumed termites and other insects. Lyke et al. (2019) reported ecological niche differences in the dietary arthropods of blue‐ and red‐tailed monkeys. DNA metabarcoding has also been reported in studies of colobine in plant diets, such as *R. brelichi* (Yue et al., 2023), *Presbytis femoralis* (Srivathsan et al., 2016) and *Pygathrix nemaeus* (Srivathsan et al., 2015). Collecting faeces does not involve coming into direct contact with the rare *R. brelichi* and does not affect their behavioural activities. Therefore, using faecal samples to study the diet of *R. brelichi* is advantageous and feasible since the species is elusive and its winter habitat at high altitudes poses difficulties in following animals and to observing foraging.

Snub‐nosed monkeys are members of the subfamily Colobinae and have an enlarged, low acid and complex multi‐chambered stomach as an adaptation to their mainly folivorous diets (Kuang et al., 2023; Yang et al., 2019). Zhang et al. (2021) reported that *Trachypithecus leucocephalus* feeds mainly on the leaves of a variety of plants throughout the year in Guangxi Province, China, possibly benefiting from the high level of dietary diversity in the area. In Angola, black and white colobus monkeys (*Colobus angolensis palliatus*) consume foliage that is high in phosphorus and low in calcium (Dunham & Opere, 2020). *Procolobus badius* and *Colobus guereza* in Kibale National Park, Uganda, prefer foods with high protein and low fibre content (Wasserman & Chapman, 2003). Asian colobus monkeys typically require extended days and overnight stays to rest and digest fibre‐rich plants (Huang et al., 2023). All species of the genus *Rhinopithecus* feed on seeds, fruits, insects, buds and bamboo (Yang et al., 2019). The diet of *R. brelichi* includes leaves (47%), buds (25%), flowers (9%), fruits and seeds (22%), bark, bulbs, animals and insect larvae (7%) (Long et al., 2022). The diet of *R. brelichi* exhibits obvious seasonality. In spring, their diet mainly consists of leaves and flowers; in summer, the diet consists of leaves and immature fruits; and in autumn, the diet consists of fruits and leaves. A total of 83.7% of the winter diet consisted of tree buds, while bark and flower buds were also consumed. Wu et al. (2004) found that the most suitable habitats for *R. brelichi* were oak forest and Magnolia forest. Niu et al. (2014) used faecal microscopy to analyse surface food use during the snow season of 2012 and reported that *R. brelichi* foraged on plant parts such as shoots, buds, leaves, flowers, bark, fruits and seeds. These studies mainly used foraging behaviour and faecal microscopy to study the food composition of *R. brelichi*. There is a lack of studies of *R. brelichi* diet composition using DNA metabarcoding and little information on use of animal prey to supplement the diet of *R. brelichi*.

Winter in subtropical regions is a period of lack of high‐quality food supply for animals, which can threaten the survival of *R. brelichi*, and the study of its winter diet can provide key information for its conservation, such as the discovery of its fallback foods. Our study aimed to address three questions: (1) What are the main dietary components of *R. brelichi* in winter? (2) What are the habits of plants consumed by *R. brelichi*? (3) Is it possible to discover new food items for *R. brelichi*? In the end, we propose the following three hypotheses: (1) *R. brelichi* consumes shrubs and herbs in winter; (2) the diversity of the plant diet is greater than that of the animal‐diet in *R. brelichi*; and (3) the existence of new dietary species in *R. brelichi* has yet to be discovered. In this paper, we used a faecal DNA metabarcoding approach to study the dietary composition of *R*. *brelichi* in winter and the diversity of the plants and animals consumed in the Fanjingshan Nature Reserve. By analysing the faecal samples of *R. brelichi*, our objectives were (i) to learn about their foraging preference in winter, (ii) to discover the habit of plant food in winter, and (iii) to find new foodstuffs that have not yet been reported.

## 2 MATERIALS AND METHODS

We obtained permission to conduct this study from the FNNR Administration in Guizhou, China. This project complies with all necessary legal requirements stipulated in the Wildlife Protection Law of the People's Republic of China (NPC, 2022). We have no direct interaction with grey snub‐nosed monkeys that may affect their health. We collected faecal samples from forest trails and areas of high animal activity frequencies according to our previous observations.

### 2.1 Study site and sample collection

The FNNR (27°46′50″–28°1′30″ N, 108°45′55″–108°48′30″ E) is in Guizhou Province, Southwest China, at an altitude of 500–2572 m, and an area of 419 km^2^. The vegetation types are evergreen broad‐leaved forests, mixed evergreen deciduous broad‐leaved forests, deciduous broad‐leaved forests, mossy dwarf forests, subalpine coniferous forests and subalpine scrub meadows, in descending order of elevation. *R. brelichi* is found only in the reserve, mainly inhabiting deciduous and evergreen broad‐leaved mixed forest belts (Xiang et al., 2009). In addition, there are herbivores such as *Capricornis milneedwardsii*, *Elaphodus cephalophus*, *Muntiacus reevesi*, *Sus scrofa* and *Hystrix hodgsoni*, and primates such as *Tibetan macaques* and *Macaca mulatta* are present in the reserve (Wan et al., 2021; Xie et al., 2022). There are also carnivores and omnivores such as *Paguma larvata*, *Prionailurus bengalensis*, *Viverricula indica*, *Prionodon pardicolor*, *Arctonyx collaris*, *Meles meles*, *Melogale moschata*, *Herpestes urva* and *Ursus thibetanus* (Wang et al., 2020, 2023). The Fanjing Mountain Nature Reserve has implemented an ecological migration and relocation policy, and residents of villages in the northern part of Fanjing Mountain have gradually moved out of the reserve. The traces of Guizhou golden monkeys have been found in these low‐altitude villages. Yang et al. (2023) have also discovered through the use of infrared cameras that Guizhou golden monkeys are active in the Fanjing Mountain ecotourism area; however, most related studies suggest that tourism facilities can hinder the migration of Guizhu golden monkeys (Ju & Dong, 2022; Wang et al., 2022).

**FIGURE 2:**
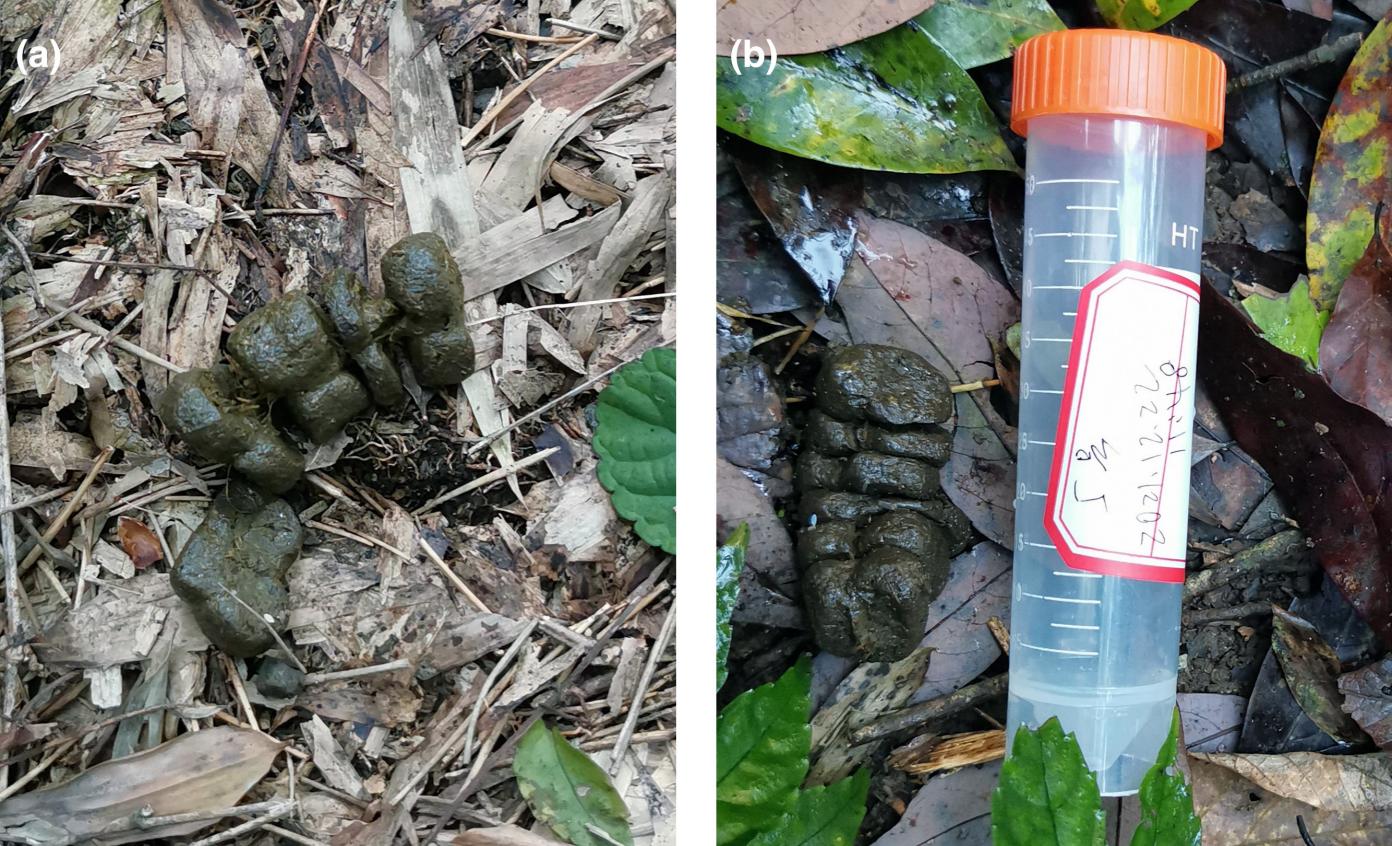
Guizhou golden monkey faeces in the field (a, b) and with 50 mL sample tubes for reference (b).

From December 2021 to January 2022 in the winter season (Nie et al., 2009), all samples of suspected Guizhou golden monkey faeces with good moisture retention, low hardness and intact, and bead‐like morphology were collected (Figure 2). The samples were picked up using disposable medical film gloves, preserved in 50 mL centrifuge tubes with anhydrous ethanol, brought back to the laboratory and stored at −80°C. Information on the latitude and longitude of the collection site and the type of vegetation was recorded.

### 2.2 DNA extraction

The faecal samples were quantified, and total DNA was extracted using an OMEGA kit (Omega Bio‐Tek, USA) according to its instructions. The DNA and RNA were analysed using 0.8% gel electrophoresis and a NanoDrop™ One/OneC Microvolume UV–Vis spectrophotometer (NanoDrop™ One/OneC Microvolume UV–Vis Spectrophotometer, Thermo Fisher Scientific, USA) to calculate A_260_/A_280_ values and to check the purity and concentration of DNA and RNA, followed by storage of DNA at −20°C.

### 2.3 PCR amplification and high‐throughput sequencing

The primers ZlaF (5′‐ATGTCACCACCAACAGAGACTAAAGC‐3′) and hp2R (5′‐CGTCCTTTGTAACGATCAAG‐3′) (Hofreiter et al., 2000) were selected for amplification of the *rbcL* region of food chloroplasts. The primers *COI* inTF (5′‐GGWACWGGWTGAACWGTWTAYCCYCC‐3′) and *COI* jgHC02198 (5′‐TANACYTCNGGRTGNCCRAARAAYCA‐3′; Hawke et al., 2022) were selected for amplification of mitochondrial *COI* genes. The total volume of the PCR system was 25.00 μL, which included 5.00 μL of 5× Reaction Buffer and 5× High GC Buffer, 0.5 μL of dNTP (10 mmol/L), 1.00 μL of each primer (10 μmol/L), 1.00 μL of DNA template, 11.25 μL of nucleic acid‐free water and 0.25 μL of Q5 high‐fidelity DNA polymerase. The PCR procedure was as follows: predenaturation at 98°C for 5 min; 30 cycles of denaturation at 98°C for 30 s, annealing at 50°C for 30 s and extension at 72°C for 30 s; extension at 72°C for 5 min; and holding at 4°C. PCR amplification products were detected by 2% agarose gel electrophoresis and recovered by purification with an Agencourt AMPure Beads Kit (Beckman Coulter Ltd.) The PCR amplification products were quantified using the PicoGreen dsDNA Assay Kit (Ingenious Life Technologies, Inc., USA). Each faecal sample was sequenced. Sequencing was performed by an Illumina NovaSeq PE250 (2 × 250 bp) (Shanghai Personalbio Biotechnology Co., Ltd., China).

### 2.4 Data analysis

We performed DNA identification of the species from which the faeces originated and discarded faecal samples from which the species could not be identified. First, the sequence files were quality checked by FastQC v0.11.9 (https://www.bioinformatics.babraham.ac.uk), and then Trimmomatic 0.39 (https://github.com/usadellab/Trimmomatic) was used for sequence quality filtering, and the final sequence length was required to be ≥150 bp. Finally, the sequence was spliced and removed using Flash 1.2.11 (https://ccb.jhu.edu) and QIIME2 2019.4 (http://qiime2.org) for sequence splicing and chimaera removal. The sequences were merged and operationally classified based on 97% sequence similarity using QIIME2.

An operational taxonomic unit (OTU) is a group of closely related individuals that are grouped together based on the similarity of specific sequences (Ciuffreda et al., 2021). The representative sequences from each OTU were annotated using BLASTN against the NCBI taxonomy database, providing classification information at various levels such as the phylum, genus, and species levels. Finally, the annotated species were manually collated with information on species distributions in FNNR (Lan & Yang, 1990; Zhou et al., 1990) to obtain the species composition and relative abundance distribution of each sample at the taxonomic level, including family, genus and species. The %RO was used to estimate the dietary composition of the animals, indicating the share and importance of a particular food OTU in the diet (Gómez‐Ortiz et al., 2015; Kasper et al., 2016; Wang et al., 2014). The %RO is the percentage of occurrence of a particular food genus or family as a percentage of the total occurrence of all food OTUs and is calculated as follows:

(1) $$\%\mathrm{RO}=\frac{N_{i}}{\sum N_{i}}\times 100\%$$

where *N*_*i*_ denotes the number of samples in which a food genus or family of type *i* occurred (number of times) and $\sum N_{i}$ denotes the sum of all food genus or family occurrences of the species. Interpolation and extrapolation curve analysis was performed with the iNEXT package and the Hill number was calculated (Hsieh et al., 2016). We selected the top 20 plant genera in terms of abundance and occurrence, plotted the percentages, categorized the genera into the corresponding families and orders according to the angiosperm classification system, and plotted the order‐family‐genus hierarchical relationships to show the occupancy and subordination of the genera in the plant orders and families. Additionally, we categorized the plant genera according to their habits, such as herbs and shrubs, as a way of revealing the main ecological habits of the plant foods. The sunburst chart and the proportion diagrams were plotted using Origin 2022.

## 3 RESULTS

### 3.1 Classification and taxonomic identification of OTUs

The 31 samples were sequenced by Illumina HTS, and 2,418,274 target fragments with an average length of approximately 202 bp were obtained after amplification of the chloroplast *rbcL* gene. The lowest number of sequences in the samples was 49,561, and the highest was 98,503, with an average sequence of 78,167 ± 10,393. The mitochondrial *COI* amplification yielded 3,105,612 target fragments with an average length of approximately 313 bp, with a minimum of 72,571 and a maximum of 161,901 sequences in the sample and an average sequence of 100,155 ± 17,203.

### 3.2 Analysis of plant food families and genera

**FIGURE 3:**
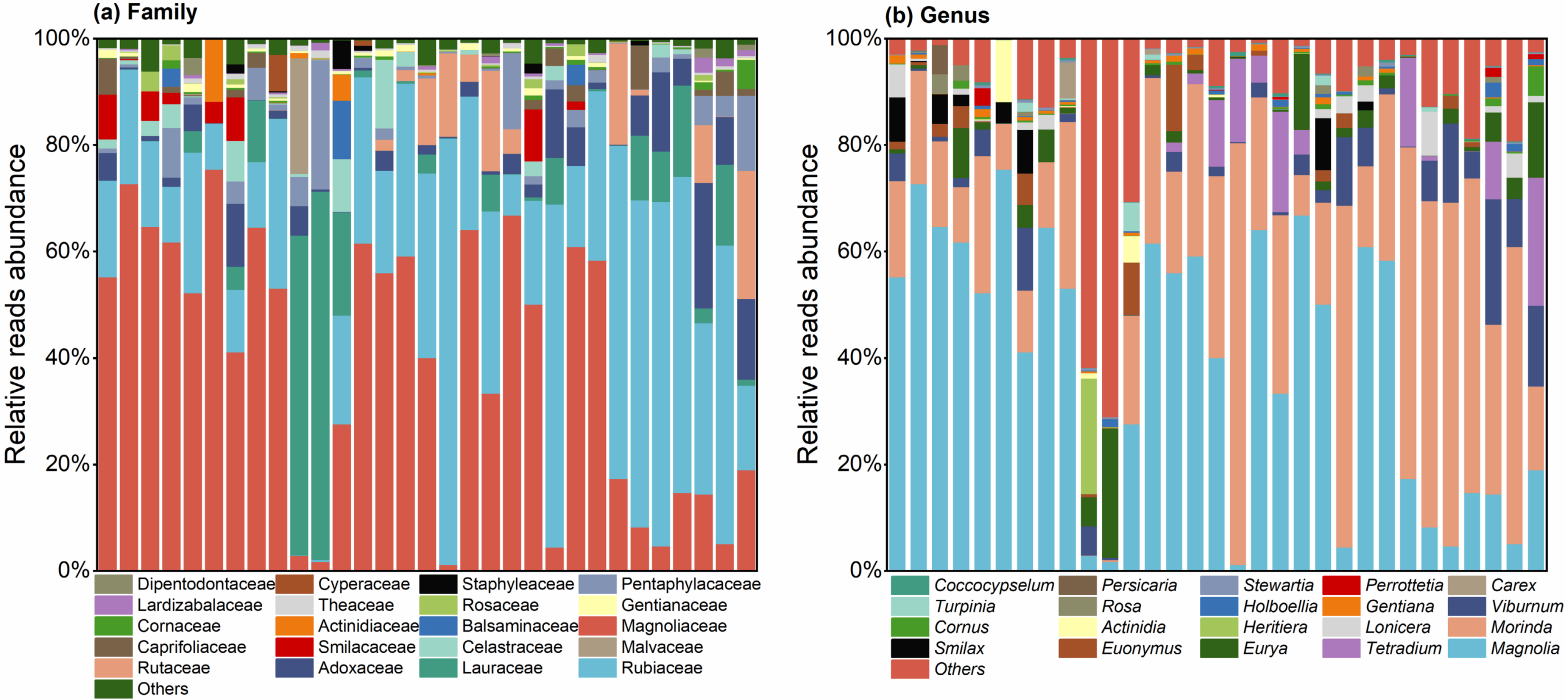
(a) Top 20 plants according to relative abundance at the family (a) and genus (b) levels for *R. brelichi* (each bar represents the contribution of each plant family or genus in the contents of each faecal sample).

Thirty‐one faecal samples were identified, and the families and genera with the top 20 relative abundances of *R. brelichi*‐fed plants were selected for presentation (Figure 3), while families and genera with relative abundances outside the top 20 were combined into the Others category. At the family level, Magnoliaceae (1.16%–75.41%), Rubiaceae (0.1%–80.08%), Lauraceae (0.01%–69.16%), Adoxaceae (0.01%–23.54%), Rutaceae (0–24.07%), Pentaphylacaceae (0.016%‐ 24.30%), Celastraceae (0–12.87%), Smilacaceae (0–9.75%) and Caprifoliaceae (0–8.23%) had mean relative abundances greater than 1%. At the genus level, *Magnolia* (1.16%–75.41%), *Morinda* (0.072%–79.25%), *Viburnum* (0.01%–23.54%), *Tetradium* (0–24.06%), *Eurya* (0.016%–24.29%), *Euonymus* (0–12.43%), *Smilax* (0–9.75%) and *Lonicera* (0–8.23%) had average relative abundances >1.0%.

**FIGURE 4:**
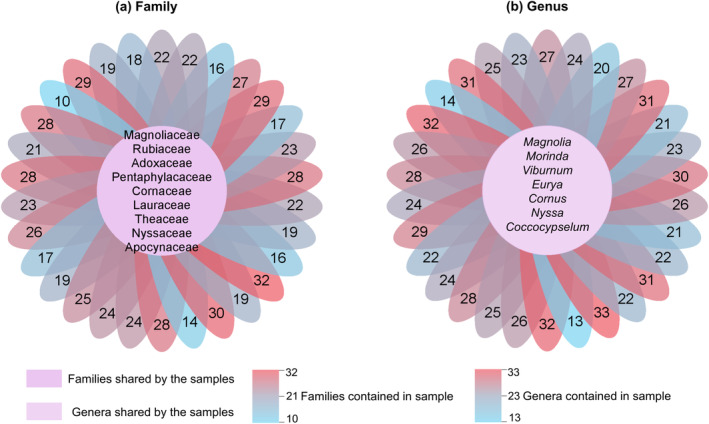
Flower plots of plant family (a) and genus (b) that overlapped in all samples. They illustrate the number of plant families or genera contained in each sample (in the petals), as well as the plant families or genera shared by the sample (in the centre).

Sample analyses revealed the overlap of plant families and genera (Figure 4). The Magnoliaceae, Rubiaceae, Adoxaceae, Pentaphylacaceae, Cornaceae, Lauraceae, Theaceae, Nyssaceae and Apocynaceae were the families present in all 31 samples; Gentianaceae, Aquifoliaceae, Symplocaceae, Araliaceae, and Rosaceae were the families present in all but one sample. *Magnolia*, *Morinda*, *Viburnum*, *Eurya*, *Cornus*, *Nyssa* and *Coccocypselum* were the genera present in all 31 samples; *Gentiana*, *Ilex* and *Symplocos* were the genera present in all but one sample; *Stewartia* and *Camellia* were present in 29 samples.

### 3.3 Analysis of animal and macrofungal food families

**FIGURE 5:**
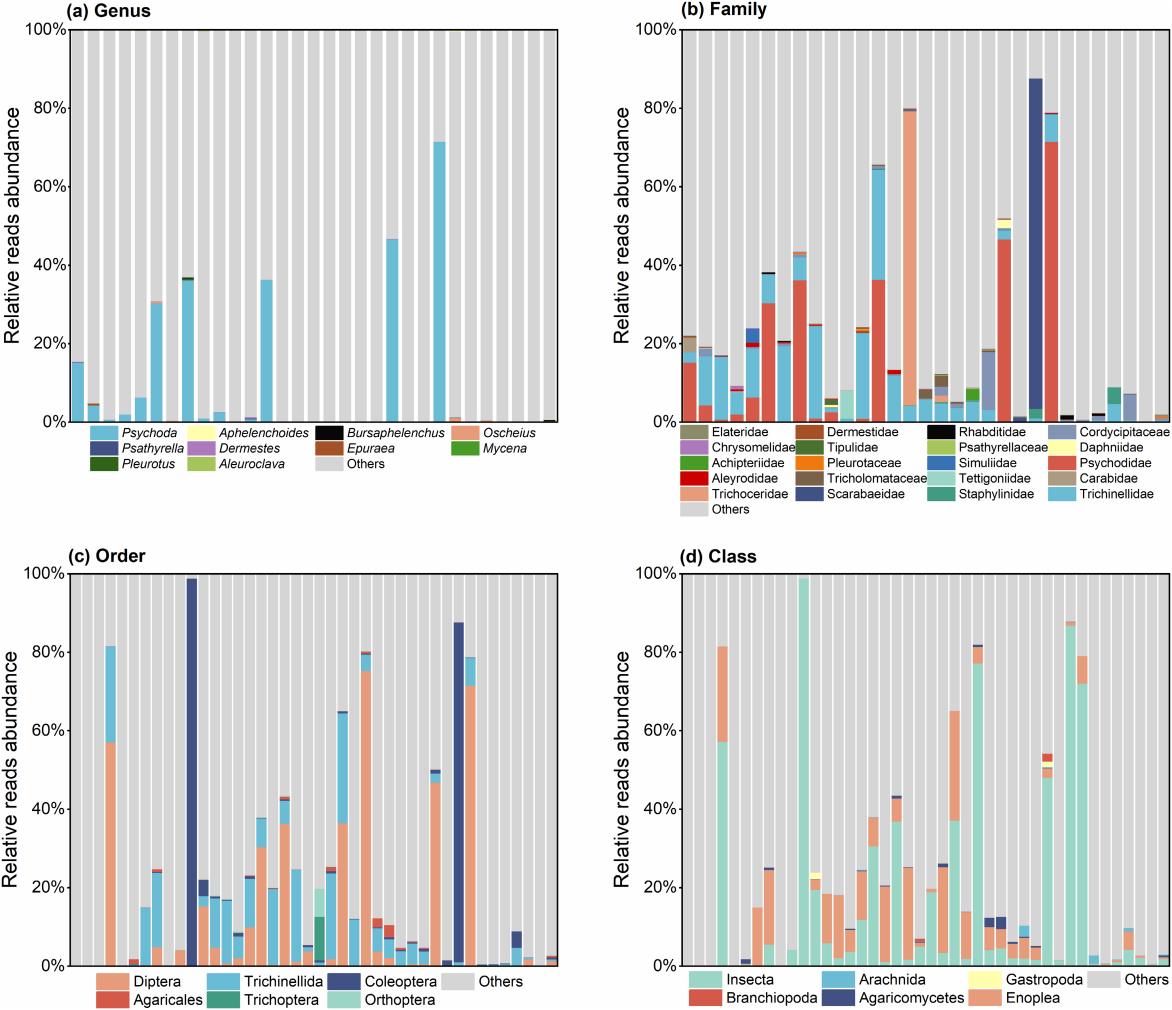
Top 10 (a), top 20 (b), top 6 (c) and (d) animal and macrofungal foods according to relative abundance at the genus, family, order and class levels respectively, for *R. brelichi*.

As shown in Figure 5, for the animals identified, at the family level, Psychodidae (0–71.51%), Trichinellidae (0.6%–27.97%), Staphylinidae (0–4.20%), Scarabaeidae (0–84.13%) and Trichoceridae (0–74.81%) had an average relative abundance greater than 1%; the top genera in term of relative abundances were *Psychoda* (0–71.51%), *Oscheius* (0–1.11%) and *Dermestes* (0–0.39%). At the animal order and class level, Diptera (0–75.24%), Trichinellida (0–27.97%), Coleoptera (0–98.81%), Insecta (0.01%–98.84%) and Enoplea (0–27.97%) were dominant. *Pleurotus* (0–0.49%), Cordycipitaceae (0–14.75%), Agaricales (0–3.13%) and Agaricomycetes (0–3.13%) were more dominant in the macrofungal diets.

### 3.4 Analysis of diet composition diversity

**FIGURE 6:**
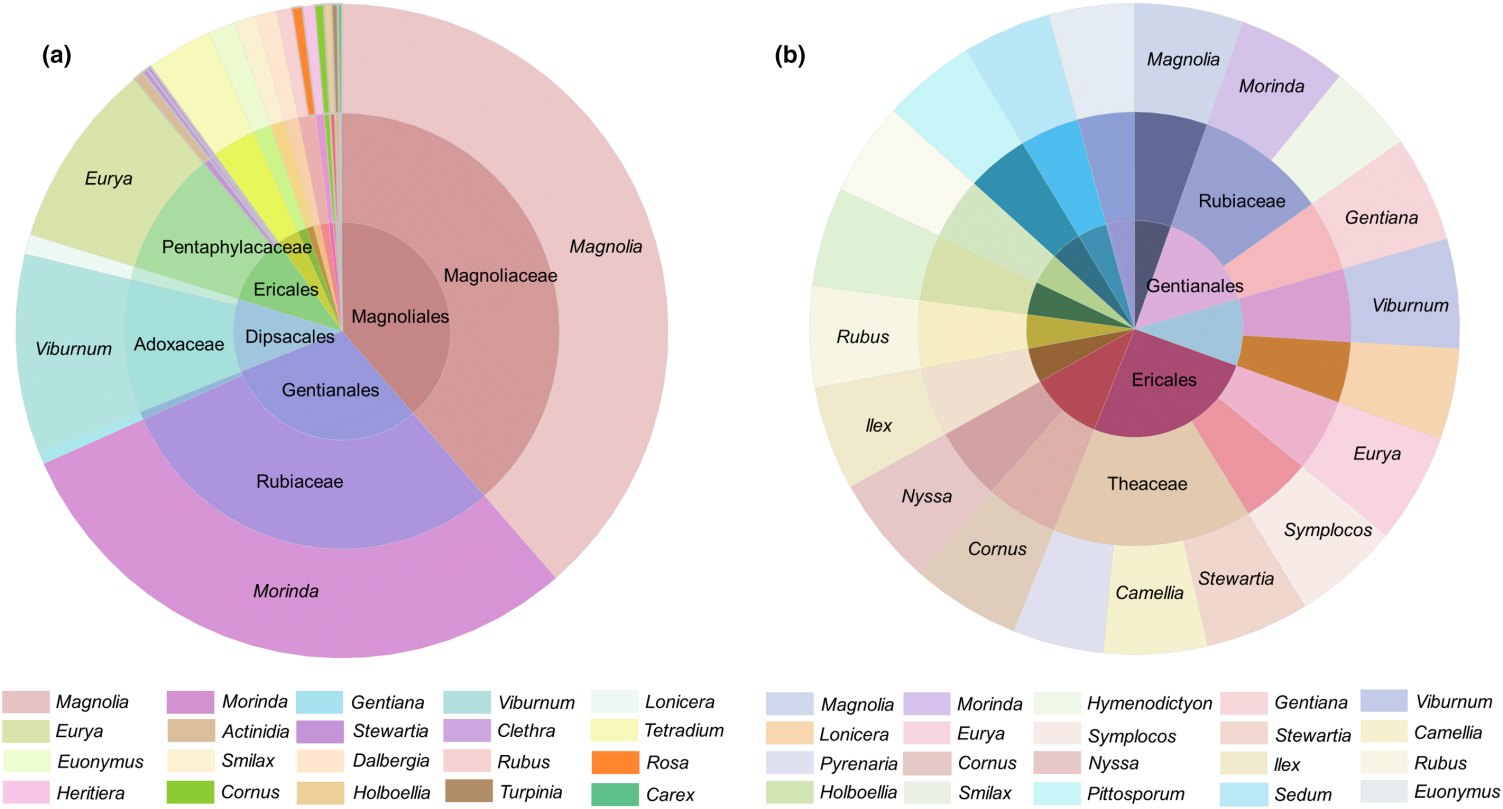
Sunburst chart of the top 20 genera according to relative read length abundance (a) and percentage of occurrence (%RO) (b) of plant food (the innermost circle indicates the order, the middle circle indicates the family and the outermost circle indicates the genus).

The top 50 genera, in terms of the relative abundance of plant food components, were selected, and the relative abundance was normalized to those with a normalized value greater than 0.5% for family and order grouping (Figure 6a). The dominant orders and families were found to be Magnoliales, Gentianales, Dipsacales, Magnoliaceae, Rubiaceae, Adoxaceae and Rutaceae. The percentage of occurrence (%RO) of food genera in all samples was calculated (Figure 6b), and the dominant orders and families were found to be Ericales, Gentianales and Theaceae.

**FIGURE 7:**
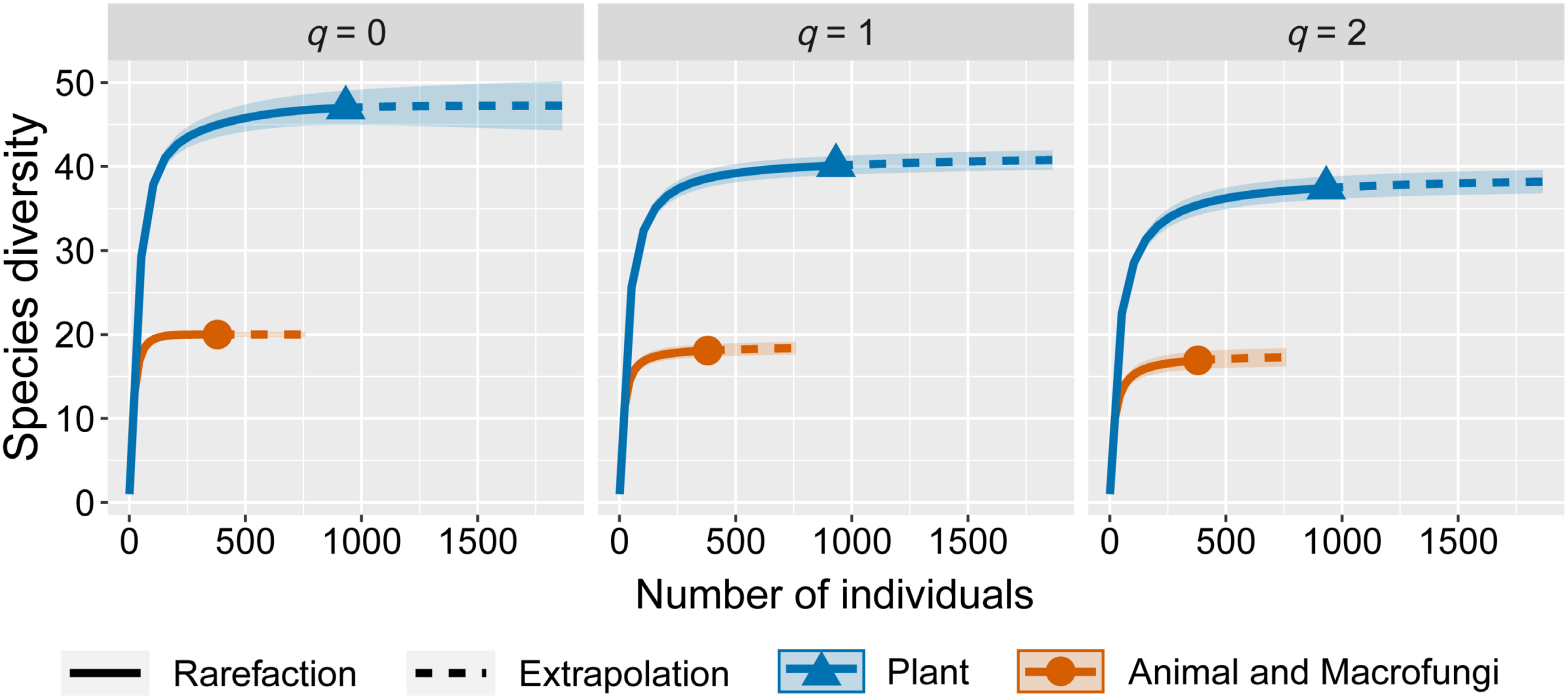
Interpolation and extrapolation curves of plant, animal and macrofungal diets in the faeces of *R. brelichi* using iNEXT; the solid line represents interpolation curves, and the dotted line represents extrapolation curves; shaded areas represent 95% confidence intervals, separated by diversity order: *q* = 0 (species richness, left), *q* = 1 (Shannon diversity, middle) and *q* = 2 (Simpson diversity, right).

As shown in Figure 7, the species richness values of the *R. brelichi* plant, animal and macrofungal diets were 47 and 20, respectively, with Shannon diversity values of 40.12 and 18.09 and Simpson diversity values of 37.44 and 16.92, respectively. These findings suggested that the number of species, the effective number of common species and the effective number of dominant species in the *R. brelichi* plant diet were greater than those of animals and macrofungi.

### 3.5 Plant food habit analysis

**FIGURE 8:**
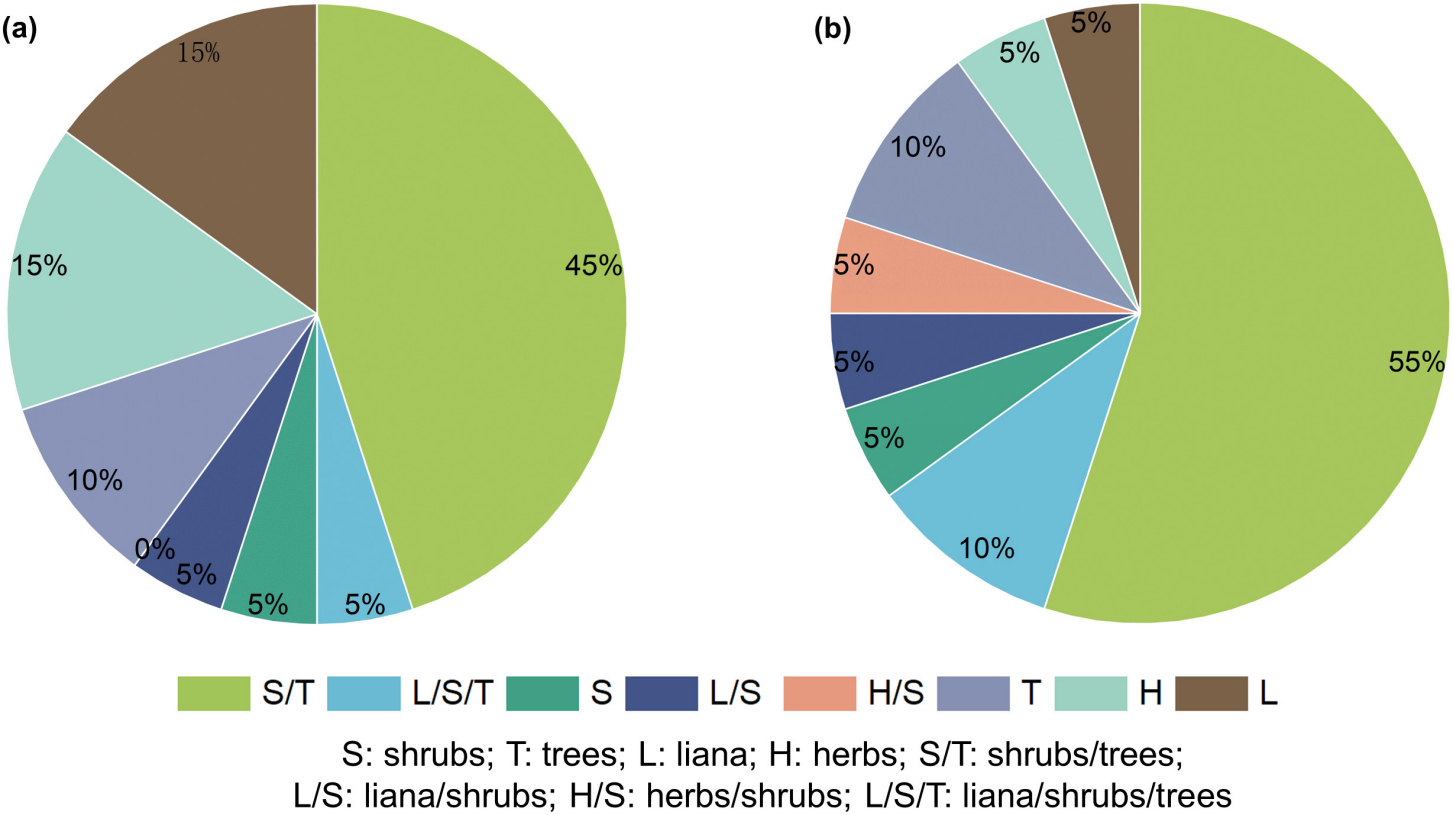
Proportion of each habit in the top 20 plant genera in terms of relative abundance (a) and percentage of occurrence (b).

The top 20 plant genera in terms of relative abundance and frequency were analysed for habit, and they were classified as herbaceous herbs (H), liana (L), shrubs (S), trees (T) or cross‐habit (Figure 8). At the abundance level, the plant habit was dominated by shrubs/trees (45%), herbs (15%) and lianas (15%); at the percentage of occurrence level, the plant habit was dominated by shrubs/trees (55%), trees (10%) and liana/shrubs/trees (10%).

## 4 DISCUSSION

This study reported the dietary diversity of wild *R. brelichi* in the FNNR, China, using DNA metabarcoding. We found that the winter diet of *R. brelichi* focused on Magnoliaceae, Rubiaceae, Adoxaceae, Diptera, Coleoptera, Trichoptera and Agaricales. *R. brelichi* is one of the most endangered primates. The DNA metabarcoding technique enables us to discover new food items that we have not yet recorded, such as *Glyptostrobus pensilis* and *Menyanthes trifoliata*, to find the main dietary plants in winter, and thus to protect the plant resources in the habitat and to provide guidance for the conservation of the habitat.

We found that the top‐ranked abundances of Magnoliaceae, Theaceae, Rosaceae, *Viburnum*, *Cornus*, and Nyssaceae have all been reported in previous *R. brelichi* dietary studies by traditional dietary analysis methods (Niu et al., 2014; Xiang et al., 2012; Yang et al., 2010). *Eurya*, *Magnolia*, *Rubus* and *Morinda* were reported to be present in the *R. brelichi* diet by DNA metabarcoding (Yue et al., 2023). In this study, Magnoliaceae was the top‐ranked family in terms of the abundance of plant food species in *R. brelichi*, with a relative abundance of approximately 40%. *Magnolia* leaves are rich in vitamins, amino acids and various trace elements. *Morinda* of Rubiaceae was the second most abundant plant in the study. *Morinda pubiofficinalis* and *M. hupehensis* are both distributed in Guizhou Province and are vines that grow in valleys, mountain forests, streams and thickets; thus, they may also constitute the main food sources for grey snub‐nosed monkeys. *Viburnum* of Adoxaceae is mostly shrubs, and its leaves and seeds are also preferred food (Niu et al., 2014; Xiang et al., 2012; Yang et al., 2010). *Tetradium* of Rutaceae was the fourth most abundant genus in this study, with *Tetradium ruticarpum* and *Tetradium glabrifolium* distributed in Guizhou Province, and these species are very likely to constitute winter food. Guizhou golden monkeys consume trees and lianas throughout the year (Xiang et al., 2012). We found that they consumed mainly shrubs and shrubs/arbours in winter through plant food habit analysis. According to the optimal foraging theory, primates may change their food choices when their preferred food is less available, causing them to make decisions based on the relationship between the energy obtained from alternative foods and the energy needed to obtain and process them (Martinez et al., 2022). When preferred foods are scarce, one strategy used by primates is to switch to lower quality or less desirable alternatives, i.e., fallback foods (Miller et al., 2020). Therefore, winter plant foods consumed by *R. brelichi* are dominated by shrubs, such as *Morinda*, *Viburnum*, *Lonicera* and *Rubus*. These results support our first hypothesis. Moreover, our results showed that plant food can be identified at the species level based on chloroplast DNA amplification. However, the abundance of sequences annotated to the species level resulting from HTS was relatively low, and in this study, the top‐ranked species in terms of abundance of the plant food composition of *R. brelichi* were *Holboellia latifolia*, *Persicaria maculosa*, *Cinnamomum camphora*, *Glyptostrobus pensilis*, *Smilax trinervula*, *Aucuba japonica*, *Camptotheca acuminata* and *Menyanthes trifoliata*. Among them are species that have not yet been reported as food for *R. brelichi*, which is in line with the third hypothesis; however, DNA metabarcoding for identification to the species level of faeces needs to be further improved. DNA barcoding is subject to the presence of pseudogenes and heterozygotes, which in turn reduces the accuracy of DNA barcoding and increases the complexity of the database. The presence pseudogenes can lead to the misclassification of a single species into multiple species (Antil et al., 2023), which reduces the abundance of each species at the match‐to‐species level. In addition, the use of a reference library for comparison can also affect the accuracy of the comparison at the species level (Keck & Altermatt, 2023). Future research could employ metagenomics, adopt clustering criteria for amplicon sequence variant (ASVs) and create local reference libraries of flora and fauna for species in protected areas to improve the level of identification of species.

Edible insects are an important source of energy, protein and fat in the primate diet (Lange & Nakamura, 2023). Despite their small size, insects are rich in high‐quality protein; essential amino acids make up more than half of their protein content (Rothman et al., 2014). In this study, *R. brelichi* was found to consume insects of Diptera, Coleoptera, Trichoptera, Orthoptera and Hemiptera, mainly in the families Psychodidae, Staphylinidae, Scarabaeidae and Trichoceridae. Sichuan snub‐nosed monkeys (*R. roxellana*) search for insects by stripping bark and turning over stones on the ground during foraging activities (Li, 2007). Xia et al. (2022) reported that Yunnan snub‐nosed monkeys (*R. roxellana*) consume insects, and seasonal food shortages can increase the dietary proportion of insects. The captive black snub‐nosed monkeys also prey on arthropods (Cnipsus colorantis, *Ramulus* sp., and a katydid, *Mirollia* sp.) (Yang et al., 2019). However, there is variability in the sizes of different insects; larger insects may be intentionally preyed upon by monkeys, but smaller insects may be the product of secondary predation by monkeys (e.g., larvae‐infested fruits and shoots). In addition, *Chiropotes albinasu*s preferentially selects fruits whose seeds are infested with larval insects, which are protein‐rich and relatively easy to digest (Barnett et al., 2023). Therefore, smaller Diptera, Trichoptera and Hemiptera insects may have been accidentally acquired by *R. brelichi* while eating plants or drinking water; on the other hand, larger Coleoptera and Orthoptera insects may have been intentionally preyed upon by *R. brelichi*. Based on the differences in abundance, we hypothesized that a greater proportion of *R. brelichi* winter dietary insects were acquired incidentally. In addition, insects contain vitamin B_12_, which is lacking in plant‐based diets, and their accidental or intentional consumption provides a supplementary source of vitamin B_12_ for mammals (Rothman et al., 2014). The study also revealed that *R. brelichi* consumus macrofungi from Agaricales, Psathyrellaceae and Pleurotaceae. Japanese macaques (*Macaca fuscata*) (Milner et al., 2021) and yellow baboons (*Papio cynocephalus*) (Kitegile et al., 2022) have also been recorded as foraging for fungi. Cheyne et al. (2019) reported a basis for mushroom foraging by Asian colobine using direct observation and camera traps and an increase in consumption in April and November. In the wild, *R. bieti* also feeds on fungi (Xia et al., 2022). Therefore, we surmise that mushrooms are likely to be a fallback food for *R. brelichi*. Moreover, the diet of *R. brelichi* consists of nematode animals, which are widely distributed in the soil (van den Hoogen et al., 2019). Plant‐parasitic nematodes exist in various parts of plants, including leaves and fruits (Nguyen et al., 2024). Therefore, we speculate that their consumption of nematodes may have been due to eating nematode‐infected plants or eating fruit that fell to the ground and attached to nematodes in the soil; moreover, we cannot rule out the possibility that they may have parasitic nematodes in their bodies.

The main foods consumed by Guizhou golden monkeys throughout the year are leaves, tree buds, fruits and flowers. The proportion of recorded food insects is relatively small and is greater only in spring and winter (Nie, 2009). The winter diet of this species is richer and more diverse in plant food than in animal food, which is consistent with the second hypothesis. As “leaf monkeys,” Guizhou golden monkeys prefer to feed on mature leaves and buds in winter. Insects are a supplemental component of the diet for *R. brelichi*, although insects provide essential proteins to primates with high energy per unit, capturing insects is energy expensive and the mass obtained is very small compared to that of plants (Rothman et al., 2014). Therefore, for rare wildlife such as *R. brelichi*, the focus should be on protecting plant food during seasons of food resource scarcity, such as *Holboellia latifolia*, *Persicaria maculosa* and *Cinnamomum camphora*, some of the species identified. In addition, providing herbaceous and shrubby plant foods should be a priority for habitat management conservation. Overall, additional research is needed on the diets of *R. brelichi*. Primers suitable for analysing primate diets, such as those for amplifying *ND5* and *COII*, should be used to analyse their diets in the future (Jackson & Nijman, 2020); Furthermore, DNA barcode libraries of protected species should be established to identify the dietary species of *R. brelichi* more accurately and to provide research support for species conservation in habitats.

## 5 CONCLUSION

This research reveals the food diversity of *Rhinopithecus brelichi* through DNA metabarcoding and provides initial insights into its winter diet. Our results showed that plant food can be identified at the species level based on chloroplast DNA amplification, with *Holboellia latifolia*, *Persicaria maculosa* and *Cinnamomum camphora* constituting the main winter diet of grey snub‐nosed monkeys. Overall, species of Magnoliaceae, Rubiaceae, Adoxaceae, Diptera, Coleoptera, Trichoptera and Agaricales are the main sources of food for grey snub‐nosed monkeys in winter. This diet composition study provides data to support the understanding of the feeding habits and foraging strategies of *R. brelichi*.

## AUTHOR CONTRIBUTIONS

**Xu Zhang:** Conceptualization (equal); funding acquisition (equal); writing – original draft (equal). **Huafu Zhong:** Investigation (equal); methodology (lead); writing – review and editing (equal). **Jingcheng Ran:** Conceptualization (equal); formal analysis (lead); funding acquisition (equal); writing – review and editing (equal). **Jiaxin Luo:** Formal analysis (supporting); visualization (supporting); writing – original draft (equal). **Meifeng Chen:** Visualization (lead); writing – original draft (equal). **Haibo Li:** Investigation (equal); validation (equal). **Yeying Wang:** Data curation (lead); funding acquisition (equal). **Shaochuan Cheng:** Investigation (equal); validation (supporting). **Yuying Yan:** Data curation (supporting); validation (equal). **Xiaolong Huang:** Conceptualization (equal); funding acquisition (equal); writing – review and editing (equal).

## CONFLICT OF INTEREST STATEMENT

The authors declare no conflict of interest.

## Data Availability

The dataset generated and analysed during the current study is available in the open figshare repository https://doi.org/10.6084/m9.figshare.23617506.
